# Inhibition of Influenza Virus Infection by *Lentinus edodes* Mycelia Extract Through Its Direct Action and Immunopotentiating Activity

**DOI:** 10.3389/fmicb.2018.01164

**Published:** 2018-05-29

**Authors:** Takahiro Kuroki, Sangjoon Lee, Mikako Hirohama, Tomohiro Taku, Michiko Kumakura, Takahiro Haruyama, Kyosuke Nagata, Atsushi Kawaguchi

**Affiliations:** ^1^Graduate School of Comprehensive Human Sciences, University of Tsukuba, Tsukuba, Japan; ^2^Ph.D. Program in Human Biology, School of Integrative and Global Majors, University of Tsukuba, Tsukuba, Japan; ^3^Department of Infection Biology, Faculty of Medicine, University of Tsukuba, Tsukuba, Japan

**Keywords:** LEM, inflammation, influenza virus, interferon, pneumonia

## Abstract

*Lentinula edodes* mycelia (LEM) solid culture extracts contain many bioactive compounds with diverse pharmacological activities such as antitumor, antiviral, and immunopotentiating effects. In this study, we examined the anti-influenza virus activity of LEM *in vitro* and *in vivo*. LEM directly inhibited influenza virus growth *in vitro* at early phases of infection, possibly at the entry process of viral particles to host cells. We also found that the nasal administration of LEM increased the survival rate of infected mice, and this was likely due to the direct action of LEM on the viral growth. The oral administration of LEM showed prolonged median survival time of infected mice. Histological analysis revealed that the moderate bronchiolitis was observed in infected mice by the oral administration with LEM, and the extent of alveolitis was dramatically reduced. The orally LEM-administered mice showed a rapid activation of *IFN-β* gene expression upon influenza virus infection. These results suggest that the immunopotentiation activity of LEM on type I IFN pathway represses the virus spread to distal alveolar regions from peribronchiolar regions which are primary infection sites in the mouse model. We propose that LEM has anti-influenza virus activities through the direct action on viral growth and stimulatory activity of innate immunity.

## Introduction

Various natural products have distinct anti-influenza virus activities, and traditional medicine based on those natural products has shown a potential in therapy of influenza symptoms (Wang et al., 2006). Mao-to, a Japanese traditional herbal medicine, is known to shorten the duration of fever caused by influenza A virus infection (Kubo and Nishimura, 2007). Ko-Ken Tang, a conventional Asian herbal medicine, inhibits influenza A virus infection through the repression of PI3K/Akt signaling pathway activated by influenza virus infection (Wu et al., 2011). A high molecular weight lignin-related fraction extracted from cones of *Pinus parviflora* Siebold et Zucc. suppresses the influenza viral growth by preventing viral RNA synthesis (Nagata et al., 1990; Watanabe et al., 1995). *Sanicula europaea* L. leaf extracts selectively inhibit influenza A viruses, but not influenza B viruses (Turan et al., 1996). These natural products show diverse pharmacological activities with a variety of bioactive components such as polyphenols, saponins, and alkaloids, although the inhibitory mechanism on virus infection is poorly understood.

*Lentinula edodes* mycelia is a whole extract prepared from the mycelial culture of Japanese edible mushroom, *Lentinus edodes*, grown in a solid medium of sugar-cane bagasse and defatted rice bran. LEM and its purified fractions have been shown to have antiviral activities against hepatitis C virus, herpes simplex virus, and human immunodeficiency virus (Suzuki et al., 1990; Kawano et al., 2010; Okuno and Uno, 2011; Kim et al., 2014; Matsuhisa et al., 2015). However, the antiviral activity of LEM against influenza virus remains unclear. Here we examined the inhibitory effect of LEM on influenza virus infection *in vitro* and *in vivo*. LEM directly inhibited the virus growth of influenza virus possibly by preventing the entry and/or uncoating processes of viral infection. The intranasal administration of LEM increased the survival rate of influenza virus-infected mice. In mice orally administered with LEM, although the mortality rate was not significantly reduced, the median survival time was prolonged from 9 to 13 days post infection. We also found that the oral administration of LEM reduced severe alveolitis, and the pulmonary lesion was mainly observed at peribronchiolar region of influenza virus-infected mice. The expression of *IFN-β* gene was rapidly induced in orally LEM-treated mice upon virus infection, indicating that LEM protects mice from influenza virus infection by not only the direct action on viral infection but also promoting the innate immune response.

## Materials and Methods

### Biological Materials

The powder of *Lentinus edodes* mycelia extracts was provided from Noda Shokukin Kogyo Co., Ltd., Japan. The powder of LEM was dissolved in ultra-pure water (Millipore) at a concentration of 50 mg/ml. Madin-Darby canine kidney (MDCK) cells and human embryonic kidney 293T cells were cultured as previously described (Mori et al., 2011). Female C57BL/6J mice (8–10-week-old) were purchased from CLEA Japan. All *in vivo* experiments were carried out according to the Guidelines for Proper Conduct of Animal Experiments from Science Council of Japan. The protocols for experiments with mice were approved by Animal Care and Use Committee of the University of Tsukuba.

### Virus

Influenza A/Puerto Rico/8/34 (PR8), A/WSN/33 (WSN) and B/Shanghai/361/02 (SH361) viruses were grown at 35.5°C for 48 h in allantoic sacs of 11-day old embryonated eggs, and then the infected allantoic fluid was collected and stored at -80°C until use. The viral titer of each virus measured by plaque assays was 2.0 × 10^8^ pfu/ml for PR8, 2.8 × 10^8^ pfu/ml for WSN, and 5.1 × 10^6^ pfu/ml for SH361, respectively.

### Plaque Assay

A confluent monolayer culture of MDCK cells (1.0 × 10^6^ cells) in a 6-well tissue culture plates was washed with serum-free MEM and then was infected with 50 pfu of WSN or SH361. After virus adsorption at 37°C for 1 h, the cells were washed with serum-free MEM and then overlaid with MEM containing 0.8% agarose, 0.2% BSA, 1× vitamin solution (Gibco), and 1 μg/ml TPCK-treated trypsin (Sigma) in the presence or absence of 300 μg/ml of LEM. After incubation at 37°C for 2–3 days, cells were fixed with ethanol-acetic acid (1:1) solution and stained with 0.5% amide black. To quantify the viral growth, the area of each plaque was measured by ImageJ software (NIH). Results were represented as a ratio of the plaque area formed in the presence of LEM to that in the absence of LEM.

### *In Vivo* Infection Experiment

Mice were anesthetized using isoflurane diluted at 30% in propylene glycol prior to infection. For the nasal administration of LEM, mice were infected intranasally with 1,000 pfu of PR8 in 50 μl of PBS with or without 1 mg/ml LEM. For the oral administration of LEM, mice were infected intranasally with 1,000 pfu of PR8 and then administered with 1 mg/ml LEM through drinking water during the whole period.

### Mini-Replicon Reporter Assay

293T cells were transfected with viral protein expression plasmids encoding PA, PB1, PB2, NP, and pHH21-vNS-Luc reporter plasmid (Obayashi et al., 2008). pHH21-vNS-Luc carries *firefly luciferase* gene in reverse orientation sandwiched between 23 nt-long 5′- and 26 nt-long 3′-termini of influenza virus genome segment 8. A plasmid encoding *Renilla luciferase* was used as an internal control. At 12 h post transfection, cells were further incubated with 300 μg/ml LEM for 6 h. The cell lysates were prepared, and the luciferase activity was measured using MiniLumat LB 9506 (Berthold).

### Histological Analysis

For lung histological analysis, mice were infected as described above and sacrificed at 7 days post infection. The lungs were fixed with 10% formalin. After paraffin embedding, mice lungs were sectioned into 2 μm slices, and the sections were then subjected to hematoxylin and eosin staining. Stained samples were observed using DMi8 light microscopy (Leica).

### RNA Analysis

Total RNAs were isolated by using RNeasy Mini Kit (Qiagen), and were reverse transcribed with following primers: 5′-GACGATGCAACGGCTGGTCTG-3′ for segment 5 viral genome, oligo(dT)_20_ for mRNA, and 5′-GGGAGTGGGTAATTTGCGC-3′ for 18S rRNA. Real-time PCR was performed using FastStart SYBR Green (Roche) with following specific primer sets: 5′-GACGATGCAACGGCTGGTCTG-3′ and 5′-AGCATTGTTCCAACTCCTTT-3′ for segment 5 and *NP* mRNA; 5′-GCACTGGGTGGAATGAGACT-3′ and 5′-AGTGGAGAGCAGTTGAGGACA-3′ for *IFN-β* mRNA; 5′-CTGCCATCAAGAGCCCCTGC-3′ and 5′-CCCTTCTCCAGCTGGAAGAC-3′ for *TNF-α* mRNA; 5′-AACGGCTACCACATCCAAGG-3′ and 5′-GGGAGTGGGTAATTTGCGC-3′ for 18S rRNA. The amounts of viral genome and mRNA were normalized by the amount of 18S rRNA, respectively.

### Flow Cytometry

Cells were collected from bronchoalveolar lavage fluid (BALF) and resuspended in RBC lysis buffer (154 mM NH_4_Cl, 10 mM KHCO_3_, and 0.1 mM EDTA) for 5 min. After washing the cells with PBS containing 2% fetal bovine serum (FBS), the cells were stained with either anti-CD45.2 or anti-IgG2a antibodies on ice for 30 min. Flow cytometry was performed using Guava easyCyto flow cytometer (Merck Millipore).

### Enzyme-Linked Immunosorbent Assay (ELISA)

The mice were infected intranasally with 1000 pfu of PR8. BALF was collected by washing the trachea and lungs by injecting 2 ml of PBS containing 0.1% BSA and was subjected to ELISA with mouse IFN-β kit according to the manufacturer’s instruction (PBL assay science).

### Focus-Forming Assay

Pulmonary viral titer of infected mouse was determined by focus-forming assay. Briefly, a confluent monolayer culture of MDCK cells in a 12-well tissue culture plate containing cover slips (4.0 × 10^5^ cells/well) was washed with serum-free Minimum essential medium (MEM; Nissui) and then was infected with homogenized lung supernatants containing viruses in serum free MEM. After incubating for 1 h at 37°C for virus adsorption, the cells were washed with serum-free MEM and then incubated for 4 h in DMEM containing 10% bovine fetal calf serum. Cells were fixed with 4% PFA for 10 min and were subjected to indirect immunofluorescence assay using anti-NP antibody as described previously described (Kawaguchi et al., 2011). The focus forming units (FFU) was determined by counting cells labeled with the fluorescent antibody.

## Results

### LEM Inhibits Influenza Virus Infection *in Vitro* and *in Vivo*

**FIGURE 1:**
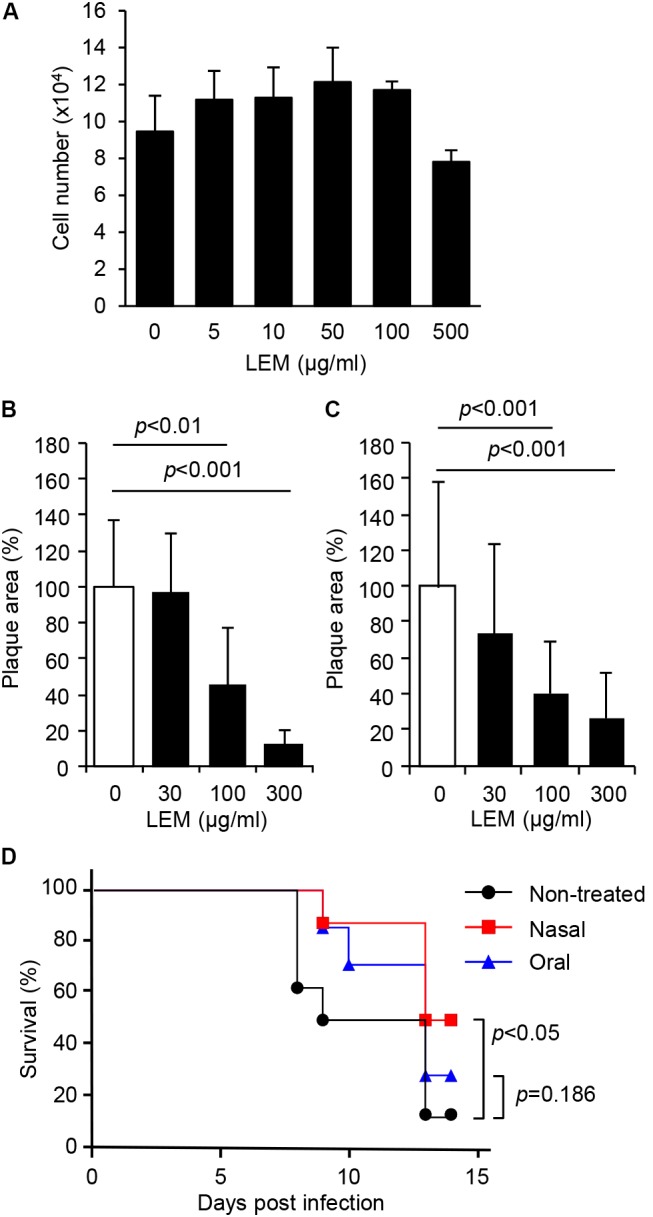
Inhibition of influenza virus infection by *Lentinula edodes* mycelia (LEM) *in vitro* and *in vivo.* **(A)** Cytotoxicity of LEM to cell growth was measured in the presence of 0, 5, 10, 50, 100, 500 μg/ml of LEM. The results are averages from three independent experiments with standard deviations. **(B,C)** Plaque assays were performed in the presence of 0, 30, 100, and 300 μg/ml of LEM with WSN **(B)** or SH361 **(C)**. The percentage of the plaque area in the presence of LEM relative to that in the absence of LEM is shown (*N* > 50). The results are averages from three independent experiments with standard deviations, and the statistical significance was determined by Student’s *t*-test. **(D)** Anesthetized mice were intranasally infected with 1,000 pfu of PR8 in the presence (Nasal in red line, *n* = 8) or absence (Non-treated in black line, *n* = 8; Oral in blue line, *n* = 7) of 1 mg/ml LEM. The mouse survival was monitored for 14 days with (Oral in blue line) or without (Non-treated in black line; Nasal in red line) the oral administration of 1 mg/ml LEM. Statistical significance was determined by Gehan–Breslow–Wilcoxon test.

To observe the cytotoxic effect of LEM, MDCK cells were incubated with MEM containing 0, 5, 10, 50, 100, and 500 μg/ml of LEM for 72 h, and then total cell numbers were counted. LEM showed little or no cytotoxicity to MDCK cells (**Figure 1A**). To next examine whether LEM inhibits influenza virus growth, plaque assays were carried out in the absence or presence of LEM. MDCK cell monolayers were infected with 50 pfu of either influenza A virus (WSN; **Figure 1B**) or influenza B virus (SH361; **Figure 1C**) and then overlaid with 0.8% agarose in MEM containing 0, 30, 100, and 300 μg/ml LEM. LEM showed a dose-dependent inhibition of plaque formation, and the area of plaques in the presence of 300 μg/ml LEM decreased to less than 15% of that in the absence of LEM (**Figures 1B,C**). These results indicated that LEM shows a broad antiviral activity against influenza viruses. We next examined the antiviral effect of LEM on survival of infected mice with either intranasal or oral administration of LEM (**Figure 1D**). For intranasal administration of LEM, fully anesthetized mice were inoculated intranasally with 1,000 pfu of influenza virus in 50 μl PBS containing 1 mg/ml LEM. For oral administration, 1 mg/ml LEM was provided through the drinking water after virus infection. The average intake amount of LEM was 11.9 ± 2.1 mg/day, and there were no signs of toxicity in uninfected mice after intranasal or oral administration of LEM. About 90% of infected mice without LEM administration died until 13 days post infection, whereas about 50% of mice intranasally administered with LEM were recovered from influenza virus infection (**Figure 1D**; *P* < 0.05). There were no significant differences in the survival rate between control and oral administration groups (*P* = 0.186). However, the median survival time of infected mice was prolonged from 9 to 13 days post infection by the oral administration of LEM, suggesting that LEM protects mice from influenza virus infection in part even through the oral administration.

### LEM Directly Inhibits Influenza Virus Infection at the Early Phases of Infection

**FIGURE 2:**
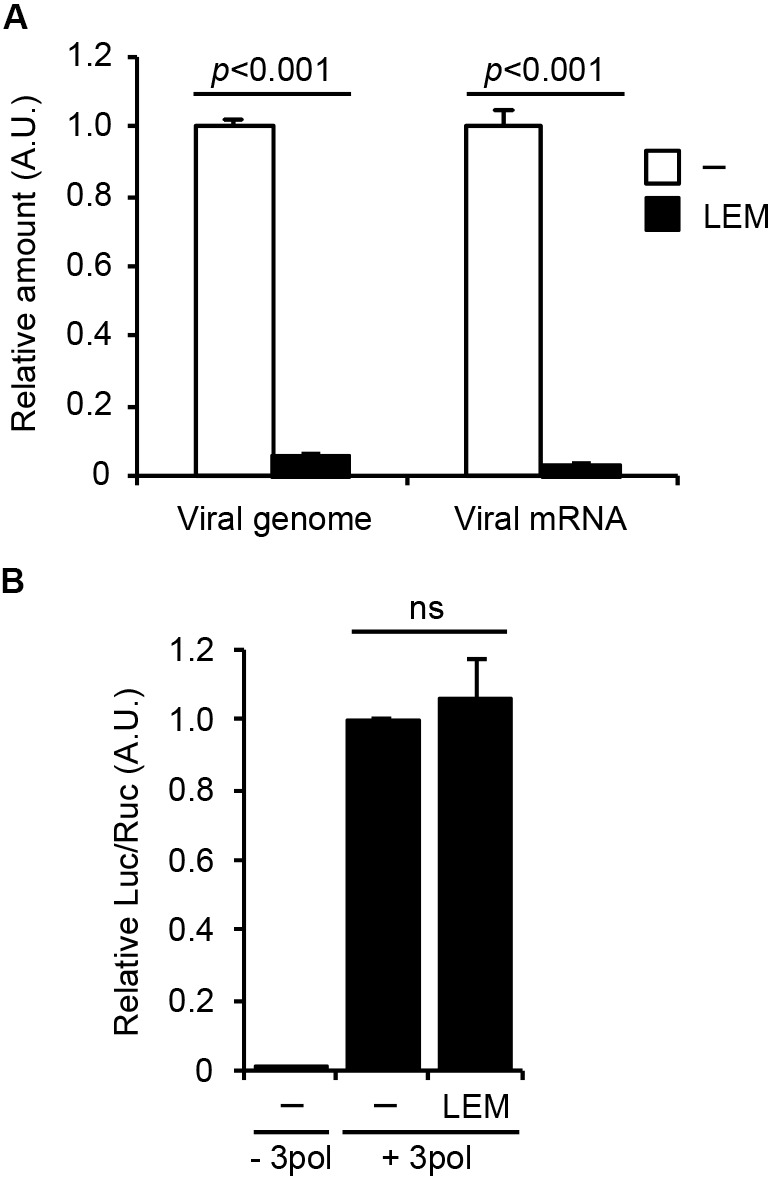
*Lentinula edodes* mycelia inhibits the early phases of influenza virus infection *in vitro.* **(A)** Madin-Darby canine kidney (MDCK) cells were infected with WSN at MOI = 3 in the absence or presence of 300 μg/ml of LEM. At 7 h post infection, total RNAs were purified and quantitatively analyzed by reverse transcription followed by real-time PCR with primers specific for segment 5 viral genome RNA and *NP* mRNA. The results are averages from three independent experiments with standard deviations, and the statistical significance was determined by Student’s *t*-test. **(B)** 293T cells were transfected with plasmids encoding *PA*, *PB1*, *PB2*, and *NP* genes and a plasmid for the expression of artificial influenza virus genome encoding *luciferase* gene as a reporter in the presence or absence of 300 μg/ml of LEM. The results are averages from three independent experiments with standard deviations, and the statistical significance was determined by Student’s *t*-test.

To address the mechanism how LEM inhibits influenza virus growth, we first examined the effect of LEM on viral genome replication and viral transcription in infected cells. At 7 h post infection, total RNAs were subjected to reverse transcription followed by real-time PCR to detect the virus genome RNA and viral mRNA (**Figure 2A**). The amounts of virus genome RNA and viral mRNA in infected cells treated with 300 μg/ml LEM reduced to less than 10% of control cells.

To further examine the effect of LEM on the viral RNA polymerase activity, we next carried out a viral model replicon assay in which active viral replicon is reconstituted with exogenously expressing viral polymerases, NP, and model virus genome containing *firefly luciferase* gene (**Figure 2B**). In this system, viral factors are expressed from plasmids, therefore we can focus on the effect of LEM on the viral polymerase activity without the entry processes of viral particles to host cells. **Figure 2B** showed no significant difference between control and LEM-treated cells in the reporter gene expression. These suggest that LEM inhibits an entry step(s) such as virus attachment and uncoating prior to the viral genome replication and transcription.

### Effect of LEM on Acute Pulmonary Influenza Virus Infection

**FIGURE 3:**
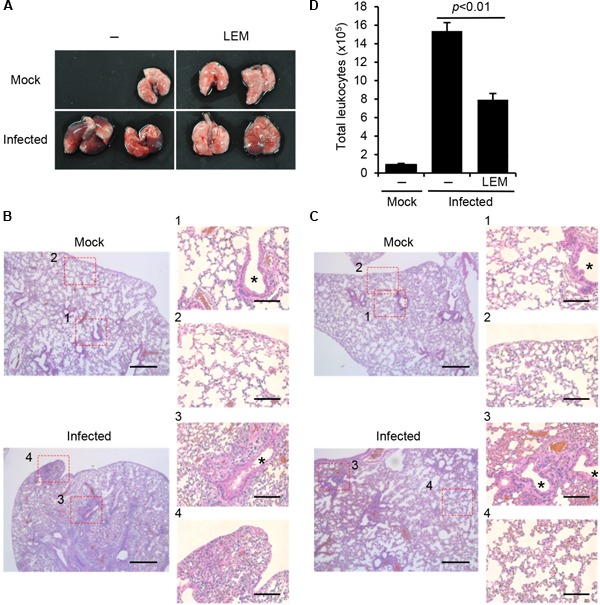
Effect of LEM on acute pulmonary influenza virus infection. Mice were intranasally infected with 1,000 pfu of PR8, and then orally administered with 1 mg/ml LEM. At 7 days post infection, the lungs of infected mice were dissected for pathological analysis. **(A)** shows the gross changes of the lungs. **(B)** (PBS) and **(C)** (LEM administration) show the hematoxylin and eosin-stained sections with enlarged figures. Asterisks indicate bronchioles. Lung section was obtained from two mice for every group and representative images are shown. Scale bar; 400 and 100 μm (in enlarged panels). In **(D)** bronchoalveolar lavage fluids (BALF) were obtained from the dissected lungs (*n* = 3 for mock-infected group; *n* = 3 for non-treated infected group; *n* = 3 for LEM-administrated infected group), and the number of leukocytes was counted by flow cytometry with anti-CD45.2 antibody, which is a leukocyte marker. The results are averages from three independent experiments with standard deviations, and the statistical significance was determined by Student’s *t*-test.

**FIGURE 4:**
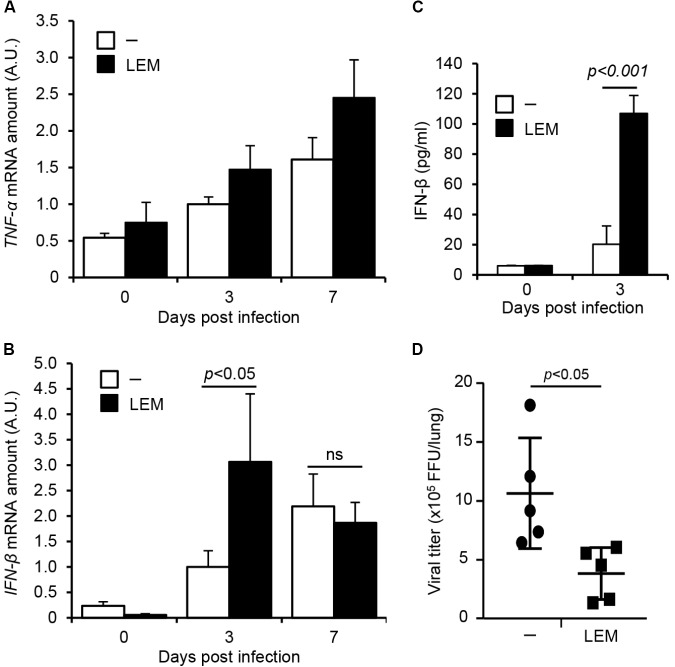
The oral administration of LEM enhances type I IFN response. Mice were intranasally infected with 1,000 pfu of PR8, and then orally administered with 1 mg/ml LEM. At 3 and 7 days post infection, total RNAs were prepared from the dissected lungs (*n* = 2 for mock-infected group; *n* = 4 for infected group) and the level of *TNF-α* **(A)** and *IFN-β* mRNA **(B)** was examined by quantitative RT-PCR. The results are averages from three independent experiments with standard deviations, and the statistical significance was determined by Student’s *t*-test. The level of IFN-β in BALF (*n* = 2 for mock-infected group; *n* = 5 for infected group) was examined by ELISA **(C)**. The pulmonary viral titers in infected mice from non-treated group (*n* = 5) and LEM-administrated group (*n* = 5) were examined by focus-forming assays at 7 days post infection **(D)**. The statistical significance was determined by Student’s *t*-test.

**Figure 1D** showed that the median survival time of infected mice is prolonged by the oral administration of LEM. To focus on the pathogenic insights, mice were infected with 1,000 pfu of influenza virus with or without the oral administration of LEM and then dissected at 7 days post infection. In orally LEM-treated mice, the macroscopic lesions with inflammation, hemorrhage, and congestion in the lungs were much smaller than those in mice without LEM administration (**Figure 3A**). We next examined histological changes of the lung slices stained with hematoxylin-eosin (**Figures 3B,C**). In the absence of LEM treatment, severe pneumonia with reduced air space of alveoli, leukocyte infiltration, and desquamation of the bronchial epithelium were observed (**Figure 3B**; enlarged figures 3, 4). In contrast, in the orally LEM-treated mice, there were infiltration with moderate numbers of leukocytes at the peribronchiolar areas (**Figure 3C**; enlarged figure 3), and the extent of alveolitis was dramatically reduced (**Figure 3C**; enlarged figures 3, 4). To quantify the level of leukocyte infiltration, we further examined the number of leukocytes into the bronchoalveolar lavage fluid (BALF) by FACS analysis using anti-CD45.2 antibody as a leukocyte marker. As expected, the number of infiltrating leukocytes in the lungs of orally LEM-treated mice decreased to about 50% of that without LEM treatment (**Figure 3D**).

To address the effect of LEM oral administration on the innate immune response, the expression levels of *TNF-α* and *IFN-β* in infected lungs were examined. At 3 and 7 days post infection, the whole lungs were dissected from infected mice orally administered with 1 mg/ml LEM. Total RNAs extracted from lungs were subjected to quantitative RT-PCR with primers specific for *TNF-α* and *IFN-β* mRNA, respectively. The oral administration of LEM led to a slight increase in the expression of *TNF-α* mRNA (**Figure 4A**). *IFN-β* was induced at 7 days post infection in non-treated mice, whereas *IFN-β* was activated at 3 days post infection in the orally LEM-treated mice (**Figure 4B**). The amount of secreted IFN-β in bronchoalveolar lavage fluid (BALF) was also measured by ELISA at 3 days post infection. The amount of IFN-β from the orally LEM-treated mice was about 5 times higher than that from non-treated mice (**Figure 4C**). This suggests that the oral administration of LEM induces the antiviral response more rapidly than non-treated mice via IFN-β production. Given the lower pathogenicity in orally LEM-treated mice, we further examined the viral titer in infected lungs at 7 days post infection. We found that the viral titer in infected lungs orally administered with LEM decreased to less than 50% of control (**Figure 4D**). These suggest that the oral administration of LEM has an immunopotentiation activity of type I IFN response to inhibit the viral growth, thereby decreases the severity of pneumonia.

## Discussion

In this study, we demonstrated that LEM inhibits influenza virus infection through the direct action at the early phases of infection, possibly at entry processes of viral particles. This direct action of LEM may contribute to improved survival rate of infected mice nasally administered with LEM. We also found that the oral administration of LEM stimulates type I IFN response upon virus infection and prolongs the median survival time of infected mice.

*Lentinula edodes* mycelia is composed of several kinds of bioactive compounds, such as polyphenolic compounds, lentinan (β-1, 3-glucan), and L2 which is a heteropolysaccharide consisting of glucose, galactose, and arabinose (Zhou et al., 2009; Xu et al., 2012). It is shown that, among polyphenolic compounds, low molecular weight lignin hydrolysates in LEM have various pharmacologic activities, such as hepatoprotective, anti-tumor, and immunomodulatory effects (Sorimachi et al., 1990; Yamamoto et al., 1997; Yoshioka et al., 2012). Our results showed that LEM directly inhibits early phases of influenza virus infection (**Figure 2**). It is reported that polyphenols in green tea have anti-influenza activity by blocking viral adsorption and entry into the host cell (Nakayama et al., 1993). It is also reported that the fruit body of *Lentinus edodes* contains lectins, which are proteins that bind to specific carbohydrates and glycoproteins (Tsivileva et al., 2001). Such lectin may inhibit the virus infection by interacting with glycoconjugated HA and/or sialic acid, which is an influenza virus receptor expressing cellular plasma membrane.

Previous studies reported that lentinan, L2, and low molecular weight lignin hydrolysates possess immunomodulatory activities (Zhou et al., 2009; Kawano et al., 2010; Xu et al., 2012). The phenolic hydroxyl group in lignin hydrolysates acts as a radical scavenger to reduce the level of nitric oxide (NO) produced from macrophages (Kawano et al., 2010). Lentinan enhances Th1 immune responses by stimulating maturation of dendritic cells to inhibit the function of regulatory T cells (Zhou et al., 2009). L2 increases the level of NO, TNF-α, and IL-6 produced from macrophages (Xu et al., 2012). In this study, we found that the oral administration of LEM stimulates type I IFN response but not proinflammatory response against influenza virus infection *in vivo* (**Figure 4**). Further, the lung inflammation was mainly observed at peribronchiolar regions but not alveolar regions of infected lungs by the oral administration of LEM. In the mouse model, influenza virus is intranasally inoculated into mice, and directly infects to bronchiolar epithelium. After the viral growth at peribronchiolar regions, the progeny viruses spread to distal alveolar regions. Thus, it is likely that the rapid activation of type I IFN pathway represses the virus spread to distal alveolar regions from peribronchiolar regions by inhibiting the viral growth at bronchiolar epithelium. It is also possible that the activation of type I IFN pathway inhibits the recruitment of neutrophils as well as the excess production of proinflammatory cytokines from macrophages that may induce tissue inflammation and lung damages (Seo et al., 2011; Xing et al., 2016). Taken together, our findings suggest that LEM has anti-influenza virus activity through the direct action on virus infection and the immunopotentiation activity of type I IFN response.

## Author Contributions

AK and KN conceived and designed the experiments. TK, SL, TT, MH, MK, TH, and AK performed the experiments and analyzed the data. TK, KN, and AK wrote the manuscript.

## Conflict of Interest Statement

The authors declare that the research was conducted in the absence of any commercial or financial relationships that could be construed as a potential conflict of interest.
